# Basal Cell Carcinoma in Asians: A Retrospective Analysis of Ten Patients

**DOI:** 10.1155/2012/741397

**Published:** 2012-07-05

**Authors:** Michael G. Moore, Richard G. Bennett

**Affiliations:** ^1^School of Medicine, Indiana University, Indianapolis, IN 46202-3082, USA; ^2^School of Medicine, University of California at Los Angeles, Los Angeles, CA 90095, USA

## Abstract

*Background*. Few studies have been done that characterize basal cell carcinoma (BCC) in Asians because this tumor is relatively uncommon in this population group. *Objective*. To characterize BCC in Asians. *Methods*. We retrospectively examined fifteen patient variables and eight tumor variables of ten Asian patients with BCC and compared these results to those of thirty matched Caucasian controls with BCC. *Results*. Asians developed their first BCC at an older age than the age of first BCC in Caucasian controls (68.9 years versus 58.3 years; *P* < 0.05). During their lifetime, Asians had fewer BCCs than the number of BCCs in Caucasian controls (1.11 versus 5.41; *P* < 0.02), despite a similar estimated lifetime daily sun exposure (hours/day) for both groups. Compared to BCCs in Caucasian controls, a higher percentage of BCCs in Asians were clinically pigmented (50.0% versus 3.3%; *P* < 0.01). *Conclusion*. Asians develop BCCs later in life and develop fewer BCCs over their lifetime than Caucasians, despite similar estimated lifetime daily sun exposure. This finding is probably due to skin pigmentation in Asians being more protective of ultraviolet light than skin pigmentation in Caucasians.

## 1. Background

Basal cell carcinoma (BCC) is the most common skin cancer in Caucasians but is uncommon in Asians (referring to the non-Indian population originating in Asia) and Black African races [1–3]. Many reviews have been published that give a clinical overview of BCC and describe its incidence in light-skinned Caucasians [4–14]. Less attention has been paid, however, looking at BCC in nonwhite races such as Black Africans [2, 3, 15–18] and Asians [1, 3, 19–30]. Those studies that have been performed on Asians were primarily done in Japanese hospitals and consequently lacked a control population that allowed for direct comparison to other races [1, 20–23, 27]. Therefore, we analyzed patient and tumor variables in our Asian patients with BCC and compared these data with those in matched Caucasian controls.

## 2. Patients and Methods

Medical records of ten sequential Asian patients with BCC from the Mohs micrographic surgery practice of one of the authors (RGB) from January 1992 to January 2001 were identified. All patient records were deidentified prior to performing a retrospective review and consequently Institutional Review Board approval was not obtained.

We recorded for analysis fifteen patient variables and eight tumor variables from these patient charts. The fifteen patient variables included the following: ethnic background, gender, age of onset of first BCC, occurrence of multiple (>1) lifetime BCCs, the number of lifetime BCCs, Fitzpatrick skin phototype, presence of fair skin, estimated average lifetime daily sun exposure (hours/day), chemical exposure history, excessive X-ray exposure history, smoking history (pack years), non-BCC skin cancer history, family skin cancer history, and personal and family history of other cancer. The eight tumor variables included the following: clinical pigmentation, duration prior to treatment, location, BCC subtype, number of Mohs stages required for removal, preoperative area (measured BCC size), postoperative area (measured postoperative wound size), and history of prior treatment.

To compare the patient and tumor variables of our Asian patients with those in Caucasians, we chose, using a table of random numbers applied to the Mohs surgery log book, thirty Caucasian control patients matched for gender and year of treatment. We excluded from this study BCCs arising in association with sebaceous nevus of Jadassohn and patients who were immunocompromised or who had basal cell nevus syndrome or xeroderma pigmentosum.

A Fitzpatrick skin phototype (i–vi) was assigned to each patient based on their ability to tan and burn from sunlight. These phototypes were determined as follows: (i) burns easy, never tans; (ii) burns easy, tans minimally; (iii) burns sometimes, tans sometimes; (iv) burns minimally, tans well; (v) rarely burns, tans well; (vi) never burns, very dark skin [31]. Besides the Fitzpatrick skin phototype, the skin of each patient was classified as fair, medium, or dark.

The number of smoking pack years was calculated as follows: mean number of packs of cigarettes smoked per day multiplied by the number of years smoked.

*P* values were calculated using the two-tailed Students *t*-test for comparison of means and the Fisher exact test for the comparison of percentages. Differences were considered statistically significant for *P* ≤ 0.05.

## 3. Results

The ethnic backgrounds and genders of the ten Asian patients are as follows: five Japanese, one South Korean, one Philippino, one Philippino/German, one Chinese/Philippino, and one Chinese patient. There were five men and five women. The ethnic backgrounds of all the Caucasian controls were European. The data for the remaining thirteen patient variables in the Asian patients and Caucasian controls are summarized in Table 1. The data for the eight tumor variables are summarized in Table 2.

The mean age of onset for the first BCC was significantly (*P* < 0.05) older for the Asian patients (68.9 years, range 43 to 85) than the mean age of onset of first BCC in the Caucasian controls (58.3 years, range 29 to 90). Also of significance was the percent of patients that had multiple (>1) lifetime BCCs, which was greater in the Caucasians than in the Asians (79.3% versus 11.1%; *P* < 0.01), and the mean number of lifetime BCCs, which was also greater in the Caucasians than in the Asians (5.41 versus 1.11; *P* < 0.02). There was a trend towards a higher percentage of Caucasians (33.3%) that had a family history of skin cancer than the percentage of Asians with a similar history (0%; *P* < 0.08). Although the *P* value for this difference was not statistically significant, this could have been due to the small number of Asians in the study.

Most Asians in our study had a medium to dark complexion. These Asian patients were much less likely to have fair skin that Caucasian controls (20.0% versus 62.1%; *P* < 0.04). The remaining patient variables were not significantly different between the Asians and Caucasians.

The percentage of BCCs in the Asians that were clinically pigmented was significantly greater than the percentage of clinically pigmented BCCs in the Caucasians (50.0% versus 3.3%; *P* < 0.01). In addition, the mean tumor duration prior to treatment was longer in the Asian patients than that in the Caucasians (50.6 months versus 22.2 months; *P* < 0.07). Although this difference did not reach statistical significance, it may be clinically important.

The biologic behavior of BCC was similar in the Asian patients and the Caucasian controls. This behavior was shown by the insignificant differences between the Asian group and the Caucasian control group with respect to BCC subtype classification, the number of Mohs stages required for BCC removal, the mean pre- and postoperative areas, and the history of prior treatment.

## 4. Discussion

In our study, we looked at Asian patients with BCC to see if the patient and tumor characteristics were different than those in Caucasians with BCC. We found equal numbers of males and females in the Asian population that developed BCC. Since we controlled for gender in the Caucasian group, there was no difference seen in the gender composition of our controls. Other studies that have been done in Asian, Black African, and Caucasian populations have varying gender results (Table 3). It is interesting to note that among the studies of Asians, the male to female ratio is about one (a similar ratio to that in our study), whereas in those looking at Black Africans, there appears to be a predominance of women and in studies of Caucasians, there appears to be a predominance of men with BCC. These differences may be related to cultural patterns of sun exposure (outdoor work, recreation, etc.). For instance, in the United States, men traditionally work more outdoors (e.g., fishing and farming), whereas women traditionally have indoor jobs. In Asia, perhaps women are as likely to work outdoors as men.

Sun damage to the skin is most likely the major cause of BCC. The amount of damage that occurs with sunlight exposure is related to the pigmentation within the skin. The clinically observed pigmentation within the skin is related to neither the epidermal thickness nor the number of melanocytes. Rather, it is related to the size, density, and distribution of melanosomes in keratinocytes [18]. In fair-skinned Caucasians, the melanosomes are small, nondense, and clustered within membrane-bound organelles. In Black Africans, large, dense melanosomes are dispersed freely within the cytoplasm. In Asians, there are small, dense melanosomes, usually clustered within membrane-bound organelles.

An increase in skin pigmentation results in reduced penetration of photons into the deeper layers of the epidermis, a finding demonstrated by Matsuoka et al. [33] who showed a reduced UVB stimulated vitamin D_3_ synthesis in Asians and Black Africans compared to Caucasians. By diminishing the amount of UV exposure, photon induced damage to the basal cells of the epidermis may be minimized, possibly leading to a reduced rate of malignant transformation.

Our Asian patients were found to develop their first BCC at a later age and were less likely to develop additional lesions in their lifetime than the Caucasian controls. This was true, despite having similar levels of sun, chemical, smoking, and X-ray exposure. These findings are consistent with the idea that Asians have skin pigmentation that, on average, is more protective of the carcinogenic effect of ultraviolet light than Caucasians [34]. In our patient population, a smaller percentage of Asian patients had fair skin than Caucasians; however, no significant difference was found in the Fitzpatrick skin phototypes of the two groups. The lack of the significant difference in the Fitzpatrick classifications may be due to patient subjectivity in obtaining this variable.

Our Asian patients had a mean age (68.9 years) of onset of first BCC that was greater than the mean ages (58.8–67.1 years) of onset of first BCC in patients from most previous studies in East Asia [22, 24, 27, 32] but was similar to the results by Goh et al. [19]. The reason for this discrepancy is not clear since the previous studies did not study exposure to different risk factors. Santa Monica is at 34 degrees north latitude, a value that is comparable to many cities in southern Japan and central China. In our study, all of the Asian subjects lived the majority of their lives in Southeast/East Asia or in southern California. As a result, the intensity of sun exposure in our Asian patients is most likely similar to that of populations in other Asian BCC studies. It is possible, however, that Asians living in southern California have occupations that reduce their duration of sun exposure (more indoor jobs).

In looking at the tumors of our subjects, a significantly greater percentage of BCCs in the Asian patients were clinically pigmented than the percentage of BCCs in Caucasian controls. This greater percentage of pigmented BCCs is in agreement with Kikuchi et al., who found tumor pigmentation to be the most characteristic clinical feature of BCC in Japanese patients compared to BCC in Caucasians [22].

It has been reported that Asian patients have an incidence of BCC ranging from 16 to 20 per 100,000 [23, 26] but has been shown to be on the rise since the 1960s [29, 35]. The incidence of BCC in Caucasians, however, has been estimated to be greater than 200 per 100,000 in females and greater than 400 per 100,000 in males [36]. As a result, physicians might be less likely to suspect a skin cancer in Asians, and Asians may be less likely to seek medical evaluation for a suspicious lesion. This lack of awareness was reflected in our data that showed tumor duration prior to treatment was greater in the Asians than in the Caucasian controls (50.6 months versus 22.2 months; *P* < 0.07). Although this difference was not statistically significant, it may be clinically important.

All Asian BCCs in our study occurred on the head and neck. In contrast, other studies from Japan and China found only 73–84% of BCCs occurring on the head and neck [1, 19, 22, 24]. The absence of BCCs in our study that occurred on the trunk and extremities is most likely due to the difference in referral patterns. BCCs referred to Mohs micrographic surgery are generally located on the head and neck.

We found that all Asian patients had a negative history of non-BCC skin cancer and a negative family history of skin cancer. Most likely this is due to the previously discussed lower incidence of skin cancer in this population.

In conclusion, BCC is a relatively uncommon skin cancer in Asians. When it does occur, it is no more aggressive than BCC in Caucasians. Physicians should be aware that BCC can occur in Asians and that early detection and treatment will result in the highest cure rate and the best cosmetic outcome.

## Figures and Tables

**Table 1:** Patient variables.

Patient variables	Asians (*n* = 10)	Caucasian controls (*n* = 30)	*P* value
Mean age (years) of onset for first BCC	68.9 ± 13.2	58.3 ± 14.0	<0.05
Occurrence of multiple (>1) lifetime BCC	1 (11.1%)*	23 (79.3%)**	<0.01
Mean number of lifetime BCCs	1.11 ± 0.33*	5.41 ± 5.03***	<0.02
Mean Fitzpatrick skin phototype	3.44 ± 1.42*	2.72 ± 1.16**	<0.15
Presence of fair skin	2 (20.0%)	18 (62.1%)**	<0.04
Mean sun exposure (hours/day)	2.5	2.77	<0.70
History of chemical exposure	4 (44.4%)*	7 (24.1%)**	<0.40
History of X-ray exposure	2 (22.2%)*	11 (31.0%)**	<0.46
Mean smoking pack years	18.8 ± 24.75	10.3 ± 18.0	<0.45
Hx of non-BCC skin cancer	0 (0%)	5 (17.2%)**	<0.32
FHx of skin cancer	0 (0%)	10 (33.3%)**	<0.08
Hx of other cancer	0 (0%)	8 (27.6%)**	<0.16
FHx of other cancer	5 (55.5%)*	21 (69.9%)**	<0.44

^∗^ *n* = 9, ^∗∗^ *n* = 29 due to unavailable data, and ^∗∗∗^ *n* = 27 due to unavailable data.

**Table 2:** Tumor variables.

Tumor variable	Asians (*n* = 10)	Caucasian controls (*n* = 30)	*P*-value
Clinical BCC pigmentation	5 (50.0%)	1 (3.3%)	<0.01
Mean duration (month) prior to treatment	50.6 ± 59.9	22.2 ± 33.5	<0.01
Tumor located on head	10 (100.0%)	29 (96.7%)	<0.07
BCC subtype			
Nodular	5 (50.0%)	15 (50.0%)	1
Infiltrative	1 (10.0%)	5 (16.5%)	1
Sclerosing	1 (10.0%)	2 (6.7%)	1
Other	3 (30.0%)	8 (26.4%)	1
Mean number of Mohs stages	2.33 ± 1.0	2.73 ± 3.2	<0.72
Mean preoperative area (mm^2^)	114.3 ± 228.4	77.0 ± 99.3	<0.50
Mean preoperative area (mm^2^)	546.3 ± 809.6*	393.9 ± 703.4**	<0.60
Prior treatment to the index BCC	2 (22.2%)*	9 (30.0%)	1

^∗^ *n* = 9, and ^∗∗^ *n* = 29 due to unavailable data.

**Table 3:** Impact of race on gender distribution of patients with cutaneous basal cell carcinoma.

Study	Male : female	Age of onset	Percent of tumors in the head and neck
Asian			
Present study	1	68.9	100
Zhang et al. [24] 1993 (Japan)	0.94	58.8	80
Ikeda et al. [1] 1989 (Japan)	1.1	N/A	73.6
Kikuchi et al. [22] 1996 (Japan)	0.97	59.1	75.3
Takenouchi et al. [27] 2001 (Japan)	0.93	67.1	N/A
Yap [32] 2010 (Malaysia)	1.05	60.9	82.8
Black African			
Mora and Burris [15] 1981 (US)	0.82	59	76.3
Abreo and Sanusi [2] 1991 (US)	0.73	68.5	88.3
Itayemi and Oluwasanmi [16] 1974 (Nigeria)	0.72	41.1	57.9
Caucasian			
Reizner et al. [25] 1993 (US)	1.93	56.5	54.7
Green [13] 1982 (Australia)	2.02	N/A	61.8
Czarnecki et al. [12] 1992 (Australia)	1.95	51.9	61.7
Roenigk et al. [11] 1986 (US)	1.15	63.8	58.8
Chuang et al. [9] 1990 (US)	0.85	64.6	83.9
Swerdlow [14] 1985 (UK)	1.2	N/A	92
Gallagher et al. [7] 1990 (Canada)	1.2	N/A	68.8
Dahl et al. [8] 1992 (Sweden)	0.97	N/A	60.2
